# Draft Genome Sequence of *Lactobacillus panis* DSM 6035^T^, First Isolated from Sourdough

**DOI:** 10.1128/genomeA.00778-15

**Published:** 2015-07-23

**Authors:** Yixin Zhu, Daiqiong Fang, Ding Shi, Ang Li, Longxian Lv, Ren Yan, Jian Yao, Dasong Hua, Xinjun Hu, Feifei Guo, Wenrui Wu, Jing Guo, Yanfei Chen, Xiawei Jiang, Xiaoxiao Chen, Lanjuan Li

**Affiliations:** a State Key Laboratory for Diagnosis and Treatment of Infectious Diseases, The First Affiliated Hospital, School of Medicine, Zhejiang University, Hangzhou, China; b Collaborative Innovation Center for Diagnosis and Treatment of Infectious Diseases, Hangzhou, China

## Abstract

We report a draft genome sequence of *Lactobacillus panis* DSM 6035^T^, isolated from sourdough. The genome of this strain is 2,082,789 bp long, with 47.9% G+C content. A total of 2,047 protein-coding genes were predicted.

## GENOME ANNOUNCEMENT

The species *Lactobacillus panis*, first isolated from sourdough with a long period of fermentation, are Gram-positive, nonmotile, asporogenous cells with a rod shape (1). *L. panis* was dominant in type II sourdoughs that were mostly used in industrial processes due to their less time-consuming one-stage fermentation process at temperatures exceeding 30°C (2, 3). It has also been reported as one of the typical lactobacilli in cereal fermentations (4, 5). In total mixed ration silages stored at high temperatures, *L. panis* seemed associated with changes in the fermentation products such as acetic acid due to differences in storage temperature (6). To date, tens of *L. panis* strains have been isolated from different sources. However, no genome of the *L. panis* species is available. Here, we present the draft genome sequence of the type strain of *L. panis*, DSM 6035, to support the understanding and applications of this species.

The genomic DNA of *L. panis* DSM 6035^T^ was isolated using a QIAamp DNA minikit (Qiagen, Hilden, Germany). The library was prepared with an Illumina TruSeq DNA PCR-Free LT library prep kit (Illumina, San Diego, CA) (7). The genome was sequenced on an Illumina MiSeq sequencer. In total, 4,742,466 paired-end sequenced reads with lengths of 300 bp were obtained, which yielded 2,845 Mb of total sequenced bases with 1,366-fold coverage. The *de novo* assembly was performed using Velvet (8), which yielded 219 contigs. The largest contig was 136,030 bp long.

The genome size of *L. panis* DSM 6035^T^ was 2,082,789 bp (2.08 Mb), with a G+C content of 47.9%, similar to the result measured by high performance liquid chromatography (HPLC) (1). The genome sequence was annotated using the Rapid Annotations Subsystems Technology (RAST) server (http://rast.nmpdr.org/) (9). The DSM 6035^T^ genome contains 2,047 coding sequences (CDS), 20 partial rRNAs, and 61 tRNAs. A total of 81 noncoding RNAs (ncRNAs) and 3 clustered regularly interspaced short palindromic repeats (CRISPR) were predicted.

A number of genes involved in sugar metabolism are found, including 6-phosphogluconate dehydrogenase, l-ribulose-5-phosphate-4-epimerase, etc., suggesting that this species is heterofermentative, which is consistent with previous discoveries (1). The genome information of this species will be useful for further studies of its applications in the field of food industry and other aspects.

### Nucleotide sequence accession number.

This whole-genome shotgun project has been deposited at DDBJ/EMBL/GenBank under the accession number LDPB00000000.
